# Editorial: Intracranial Bleeding After Reperfusion Therapy in Acute Ischemic Stroke

**DOI:** 10.3389/fneur.2021.745993

**Published:** 2021-08-31

**Authors:** Nishant K. Mishra, Richard Leigh, Bruce C. V. Campbell

**Affiliations:** ^1^Department of Neurology, Division of Stroke and Vascular Neurology, Yale University, New Haven, CT, United States; ^2^Department of Neurology, Johns Hopkins University, Baltimore, MD, United States; ^3^Department of Medicine and Neurology, Melbourne Brain Centre at the Royal Melbourne Hospital, University of Melbourne, Parkville, VIC, Australia

**Keywords:** hemorrhagic transformation, stroke, blood brain barrier, permeability imaging, endovascular treatment, hypothermia (induced)

The use of reperfusion therapy [IV thrombolysis with tissue plasminogen activator [tPA] or tenecteplase [TNK], and/or mechanical thrombectomy (MT)] in ischemic stroke patients is now an established standard of care (1, 2). The risk of intracranial bleeding after reperfusion therapy continues to be the most feared complication of reperfusion therapy [(1); also see review article by Charbonnier et al.]. In routine clinical practice, we aim to identify the occurrence of hemorrhagic transformation (HT) soon after the reperfusion therapy is offered. This is because early identification of HT may lead to precautionary steps such as stopping ongoing antithrombotic therapies, use of bleeding reversal agents and surgical interventions (1). HT appears as a hyperdensity on a non-contrast CT head and is not difficult to detect if the patient did not receive iodinated contrast previously (3). If, however, the patient did receive iodinated contrast prior to the CT head, as part of pre-treatment imaging with CT angiogram (CTA) and CT perfusion (CTP) or catheter angiography during MT, it is challenging to distinguish contrast staining from HT because both look similarly hyperdense. If an MRI cannot be obtained because of contraindications or unavailability, a typical course is to follow the evolution of the hyperdensity on the subsequent CT scans. This approach delays critical therapies such as antiplatelet agents in patients who have contrast staining but no HT. Dual Energy CT (DECT) offers a solution to this problem by exploiting the fact that the use of two X-ray radiation energies can distinguish tissue composition based on the differential Xray attenuation, including distinguishing iodine from blood (4). Three original papers examine the role of DECT in distinguishing HT from iodine contrast staining in stroke patients. Almqvist et al. found that, compared to a non-contrast CT head, DECT changed the diagnosis from intracranial hemorrhage to iodine contrast staining in about 10 percent of the stroke patients (n/N 3/31). Similarly, Liu et al. found that the conventional CT images obtained at the conclusion of MT led to the diagnosis of HT in 74.5% of the patients; this proportion, however, reduced to 10.4% when images were reviewed from the DECT. Liu et al. and Lun et al. showed that the interrater reliability for the detection of blood and/or contrast using DECT in stroke patients was fair (*k* = 0.3) with zero agreement between 18 readers. Interestingly, the intra rater agreement was higher at *k* = 0.7 in this study by Lun et al. Larger studies are needed to confirm these findings and standardize the image interpretation.

HT is classified into hemorrhagic infarction (HI) and parenchymal hematoma (PH) (3). Petechial hemorrhage without mass effect is referred as HI1 unless it is confluent which is classified as HI2 (3, 5). PH refers to HT associated with space-occupying effect (3, 5). Not every HT is of similar clinical significance, however (3, 6). It is typically the PH type HT that is clinically meaningful as it is commonly associated with clinical worsening (3). Patients with PH type HT and >30 percent involvement of the infarcted tissue are referred as PH2 type HT; these patients are typically the ones with the worst clinical outcomes (3). The classification of HT into HIs and PHs is typically done by visual inspection of the patient images (3). van Kranendonk et al. performed volumetric assessment of the HT and found that only the PH2 hemorrhage volume was associated with worse outcomes (OR 0.37; 95%CI 0.14–0.98).

Reperfusion therapy is associated with a greater risk of HT. However, this HT did not translate into increased mortality in clinical trials, an argument used to assuage the concerns of those worried about the excess bleeding risk [(1, 7–9), Charbonnier et al.]. In real world clinical practice, the goal is to prevent HT that causes clinical deterioration, mostly related to parenchymal hematoma (PH, blood clot with mass effect). If PH does occur, the goal is to rapidly diagnose it and emergently initiate therapies to prevent further clinical and/or radiological worsening (1). The two comprehensive review articles contributed by Charbonnier et al. and Maier et al. provide an excellent overview of the state of knowledge relevant to the identification and management of HT. The treating physicians are also advised to review the contemporary guidelines from relevant societies e.g., American Heart Association when needing to manage patients with post MT intracranial hemorrhage (1). The review article by Spronk et al. provides a detailed description of the post stroke inflammatory cascades which have been linked to HT. Spronk et al. discuss the therapeutic approaches to prevent HT and edema that have been previously tested in animals (e.g., Otaplimastat) and humans (e.g., NXY-059), and describe the biological pathways that can be targeted to inhibit specific inflammatory target to prevent HT. Similarly the review article by Bernardo-Castro et al. provides a detailed overview of the pathomechanism linked to the BBB permeability and its association with HT.

A survey of expert vascular neurologists found that the factors associated with the risk of HT include the volume of ischemia, previous use of antithrombotic medication, neurological severity, age, hyperglycemia at presentation, hypertension on admission, and cardio embolism (10). A secondary analysis of the WAKE-UP trial by Jensen et al. found that treatment with IV tPA, baseline NIHSS, ischemic stroke volume, baseline glucose levels, and atrial fibrillation are associated with the increased risk of HT or HI-type HT. The study also found that the patient's baseline NIHSS predicted PH-type HT in this population (Jensen et al.). It is not surprising that the survey of the expert vascular neurologists included the prior use of antithrombotic agents as one of the variables that would increase the risk of HT after MT (10). In an analysis of the NORDICTUS registry, Ramos-Araque et al. found that it was only the vitamin K inhibitors (OR 1.9, 95% CI 1.0–3.5, *P* = 0.04; *N* = 1,455) and not the newer oral anticoagulants (OR 0.3, 95%CI 0.0–2.4, *p* = 0.3) that were associated with an increased risk of symptomatic intracerebral hemorrhage in MT patients. These registry data add to the growing body of evidence in support of the safety of NOACs for secondary stroke prevention (11). Various other predictors of HT have been reported e.g., very low cerebral blood volume or significantly prolonged Tmax delay (12), Elands et al. reported that angiographic finding known as early venous filling is associated with increased risk of HT and could be used to identify high risk patients (OR 6.7, *p* = 0.005) [(13), Elands et al.].

From a biological stand point, cerebral ischemia activates the cascade of destructive processes like the progressive failure of the Na/K pumps, activation of NMDA receptors, alteration in the ionic homeostasis, buildup of acidotic environment, and the activation of inflammatory cascades [(14), Choi et al.]. The inflammatory response is characterized by the release of activated neutrophils, production of reactive oxygen species, and the expression of metalloproteinases (like MMP 9 and 2) (Spronk et al.). Post stroke inflammatory response, the ischemic cascade, and the patient specific clinical variables (e.g., the extent and degree of ischemic injury or the prior use of oral anticoagulant) interact with each other to result in the increased permeability of BBB Spronk et al., Bernardo-Castro et al. CT and MR based imaging algorithms can provide information on the degree and the extent of BBB permeability (15, 16). Arba et al. conducted a meta-analysis to study the association of BBB permeability with HT and found a significant association of BBB permeability with HT. The strength of association with HT was greater for the MRI based BBB permeability measures with the OR of 9.3 (95% CI, 3.2–27.6) which in case of CT based BBB permeability was 3.42 (95% CI, 1.62–7.23) (Arba et al.).

Despite their utility in predicting BBB disruption, these approaches have not been applied for use in routine clinical practice; the permeability imaging algorithms need to be standardized in order to permit comparability across all platforms and adoption in the routine clinical practice (Arba et al.). Additionally, analysis of BBB disruption will need to be performed in real time if it is to be used as part of clinical decision making. Heidari et al. investigated how BBB analysis may complement penumbral imaging for patients presenting in an extended time window. They found that patients with larger penumbral volumes had less disruption of the BBB, possibly explaining the low hemorrhage rates in studies that used penumbral imaging to guide thrombolysis in the extended time window (17). Their study highlights the potential value of incorporating a standardized BBB imaging protocol in routine clinical practice; however, this will require detailed validation in larger studies, standardization of the protocol, and clearance by the regulatory bodies.

Endovascular technologies have made huge strides in the last decade and have resulted in the higher recanalization rates over 80 percent (18). Even though successful recanalization can be achieved in large proportion of patients, the success with recanalization procedures has not translated into comparable improvement in the patient's outcomes (19–21). Only 25 percent of the stroke patients treated with MT are left with no disability (Rankin 0–1) (18). One of the reasons why the successful recanalization does not translate into comparably improved outcomes is that the ischemia activates a cascade of destructive processes like progressive failure of the Na/K pumps, activation of NMDA receptors, altered ionic homeostasis, local acidosis, and the activation of proinflammatory processes like the release of cytokines; [(14), Choi et al.] the restoration of blood flow into this ischemic, acidotic, brain tissue can at times be more destructive causing reperfusion injury and HT (22). Neuroprotective strategies are therefore urgently needed to immediately arrest the ischemic cascades at the stroke onset and prevent the buildup of proinflammatory and acidotic environment [(22, 23), Choi et al.]. Therapeutic hypothermia is one such strategy; however, its application in the clinical trials has been limited by the difficulty in quickly achieving hypothermia. Choi et al. report a novel approach that uses endovascular delivery of hypothermia to offer focused cooling of the ischemic brain tissue [selective endovascular brain cooling (24)] and avoids the systematic complication of systemic hypothermia like pneumonia and altered coagulability. This approach, however, needs testing in randomized controlled trials to demonstrate safety and efficacy (Choi et al.). Another potential neuroprotective agent is Magnesium (25). In an analysis of retrospective dataset of 242 patients, Cheng et al. found lower magnesium level at the stroke onset is associated with an increased risk of HT at 24–36 h after thrombolytic therapy. This relationship, however, does not hold true if the baseline magnesium levels are above a threshold of 0.88 mmol/L (Cheng et al.). They used this argument to indicate why the previous trials like FAST MAG did not show treatment response from the use of Magnesium. The magnesium levels were indeed higher than the physiological concentrations in the FAST MAG study (25, 26).

A common challenge encountered in contemporary stroke management is the risk of distal embolization of the clot that reduces the proceduralist's ability to achieve full reperfusion, perhaps more likely after IV tPA. This is particularly the case when the thrombectomy device is unable to reach the smaller distal branches of the vessel because of smaller vessel diameter (27, 28). A meta-analysis of the trial data by the HERMES collaborators reported no hemorrhagic complication and showed favorable outcomes from the MT of the M2 branches of the MCA (29). The analysis of MR CLEAN data (*N* = 1,349) showed that the thrombus location commonly changes between the CTA and cerebral angiography (*N* = 302; 22%) and the use of iv tPA is associated with an increased odds of thrombus migration (OR 2.0 95% CI 1.3–3.1) (30). In the subgroup of patients with MCA M1 occlusion, distal migration of the clot was associated with better odds of functional recovery (OR 1.5, *p* < 0.05) (30). Chang et al. report outcome data from the ischemic stroke patients (*N* = 170) with large vessel occlusion who received IV tPA plus mechanical thrombectomy (MT) or only (MT). In contrast to the recent DIRECT-MT trial (30), significantly greater proportions of the patients achieved recanalization (TICI 2b-3) from the combination therapy (tPA & MT) compared to MT alone (83 vs. 67%; *p* = 0.03) (Chang et al.). Importantly, the study found that the rate of clot migration was lower at 11% and the rate was only slightly increased in the IV tPA group (Chang et al.). Also, there was no difference in the rate of symptomatic hemorrhage between the two groups (Chang et al.). IV tPA remains standard in eligible patients in the setting of the drip-and-ship model of stroke care. Its role in patients for whom MT is immediately available may become clearer with the release of further results from randomized trials of the direct to MT strategy (SWIFT DIRECT trial, NCT03192332; DIRECT SAFE trial, NCT03494920) (30). An analysis of the MT data lodged within the STRATIS registry showed that the risk of subarachnoid hemorrhage was greater in patients with distal vessel occlusion and needing >3 passes (Lee et al.). Despite these findings, these is a degree of clinical equipoise regarding MT of the M2 occlusions, and it will be interesting to see the results of the MT trials that would target MCA M2 occlusions.

The American Heart Association recommends targeting blood pressure (BP) below 180/105 mmHg after reperfusion therapy. The ENCHANTED trial showed a significant reduction in the rate of intracranial hemorrhage when BP in the range of 130–140 mmHg was targeted within 1 h of tPA administration (OR 0.75, 95% CI 0.60–0.94, *p* = 0.01); this trial however showed no improvement in functional outcomes in the groups in which BP was targeted at <180 mmHg vs. BP 130-140 (31), Silverman et al. have contributed a comprehensive review article about the impact of cerebral hemodynamics after MT on patient outcomes. They report that ischemic stroke patients show a significant variability in the trajectory of their post MT systolic blood pressures (32). The review article indicates that the patients in each trajectory would require different BP goals (32). The presence of acutely elevated blood pressure or persistently high blood pressure in the post-MT phase is associated with significantly worse outcomes, including a higher rate of symptomatic HT (32). Acutely elevated blood pressure or persistently high blood pressure amongst successfully recanalized ischemic stroke patients reflects the presence of underlying untreated hypertension (32). A chronically hypertensive patient develops a pressure passive cerebral hemodynamic system; and restoration of blood flow in this system from successful recanalization increases the risk of HT (32). Silverman et al.'s review article highlights that there is a need to conduct trials that will test a differential response to BP management based on the observation that post MT BP oscillates within the autoregulatory limits or deviates from it (32). Cheng et al. reported that the mean, maximum, range, and the standard deviation of systolic BP measured at the conclusion of the MT were associated with PH2 type HT in ischemic stroke patients. This study suggested that in patients with successful recanalization the systolic BP should be kept to low levels, below 120 mmHg, following the conclusion of MT and fluctuations in blood pressure should be avoided (Cheng et al.; Appleton et al.) conducted a pooled analysis of the three glyceryl trinitrate (GTN) trials, ENOS, RIGHT, and RIGHT 2 (*N* = 715) (Appleton et al.). These trials mainly included patients treated with IV tPA (>99%) (Appleton et al.). The study failed to detect an association of the use of GTN with the risk of intracranial hemorrhage. However, among the patients randomized within 6 h, there was a trend toward reduced risk of HT and also improved functional outcomes(Appleton et al.).

We are delighted to present this research topic. It provides review articles that give a detailed overview of the epidemiology, pathophysiology, diagnosis, and management of hemorrhagic transformation after reperfusion therapy. It also identified important hypotheses for testing in future trials.

## Author Contributions

NM conceived of the presented research topic and invited co-editors RL and BC. NM drafted the editorial. All authors reviewed and edited the editorial and contributed to its final version.

## Conflict of Interest

The authors declare that the research was conducted in the absence of any commercial or financial relationships that could be construed as a potential conflict of interest.

## Publisher's Note

All claims expressed in this article are solely those of the authors and do not necessarily represent those of their affiliated organizations, or those of the publisher, the editors and the reviewers. Any product that may be evaluated in this article, or claim that may be made by its manufacturer, is not guaranteed or endorsed by the publisher.
